# Interrogation of glioma immune microenvironment identifies a non-canonical role for microglial Galectin-9 in tumor cell adhesion and phagocytosis

**DOI:** 10.3389/fimmu.2026.1733688

**Published:** 2026-03-24

**Authors:** Pravesh Gupta, Silvana Valdebenito-Silva, Gayatri Kumar, Prashanth Chakrapani, Shivangi Oberai, Minghao Dang, Hiroshi Katayama, Linghua Wang, Eliseo A. Eugenin, Krishna P. Bhat

**Affiliations:** 1 Department of Cancer Biology, Mayo Clinic, Phoenix, AZ, United States; 2 Department of Neurobiology, John Sealy School of Medicine, The University of Texas Medical Branch (UTMB), Galveston, TX, United States; 3 Departments of Translational Molecular Pathology, The University of Texas MD Anderson Cancer Center, Houston, TX, United States; 4 Neurosurgery, The University of Texas MD Anderson Cancer Center, Houston, TX, United States; 5 Molecular Pharmacology & Experimental Therapeutics, Mayo Clinic, Rochester, MN, United States; 6 Genomic Medicine, The University of Texas MD Anderson Cancer Center, Houston, TX, United States; 7 The University of Texas MD Anderson Cancer Center UTHealth Houston Graduate School of Biomedical Sciences, Houston, TX, United States; 8 The James P. Allison Institute, The University of Texas MD Anderson Cancer Center, Houston, TX, United States; 9 Institute for Data Science in Oncology, The University of Texas MD Anderson Cancer Center, Houston, TX, United States

**Keywords:** cell adhesion, Galectin-9, glioma, ligand-receptor interactome, microglia, phagocytosis, single cell RNA sequencing

## Abstract

Glioma-associated microglia/macrophages (GAMs) have traditionally been described as immunosuppressive. However, within their highly heterogeneous repertoire, pro-phagocytic and cytotoxic subsets with anti-tumoral properties exist. Although macrophages (MACs) can exhibit tumor-suppressive functions, their anti-glioma properties largely remain elusive. To identify anti-glioma myeloid cell effectors, we performed directionally concatenated ligand-receptor (L-R) interactome analyses from dendritic cells (DCs) and microglia (MG) to lymphoid (CD4^+^T, Tregs, CD8^+^T, NK, and NKT) cells identified by our recent single-cell transcriptomics interrogation of tumor-associated CD45^+^ leukocytes from tumor brains of eighteen isocitrate dehydrogenase (IDH)–stratified glioma patients. Within DC-specific and MG-specific interactomes, we identified *LGALS9*, which encodes Galectin-9, as a key mediator of cell–cell interactions in IDH-wild type (IDH-wt) gliomas. Spectral cytometry, immunohistochemistry, and Western blotting analyses confirmed the abundant expression of Galectin-9 in glioma-associated MG, but not in tumor cells. Furthermore, differential gene enrichment analyses revealed transcripts associated with cellular adhesion (coronin 1A, integrin) and phagocytosis (FcγR, phospholipase D, Rab family proteins, etc.) pathways that were significantly upregulated in Galectin-9^+^ compared to Galectin-9^−^ MG and MACs. Using an *ex-vivo* primary human microglia (pMG) and patient-derived glioma stem cells (GSCs) co-culture system, we evaluated the functional role of Galectin-9. Confocal imaging analyses of co-cultured pMG with GSCs revealed that siRNA-mediated knockdown or antibody-based neutralization of Galectin-9 significantly impaired pMG–GSC adhesion. In addition to reduced adhesion, phagocytosis of GSCs was dramatically attenuated across all Galectin-9 silenced or neutralized pMG. Altogether, our study underscores the unappreciated non-canonical role of Galectin-9 in MG as a regulator of glioma cell adhesion and phagocytosis that can be harnessed for glioblastoma immunotherapy.

## Graphical Abstract

**Figure:**
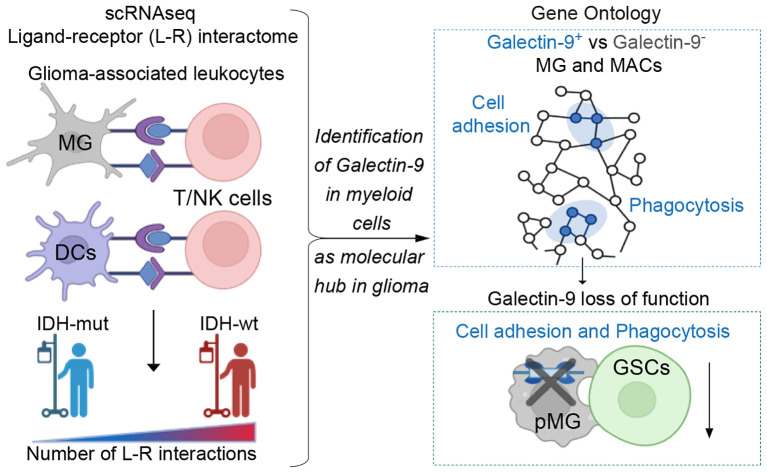

## 1 Introduction

Malignant tumors of glial origins, termed as gliomas, in particular glioblastoma (GBM), are fatal with 5-year survival rates of approximately 6% (1). Regardless of the glioma grade, about 50% of their tumor microenvironment (TME) is composed of phenotypically and functionally heterogenous brain-resident microglia (MG), macrophages (MACs), and infiltrative monocytic derivatives (2, 3), collectively referred to as glioma-associated microglia/macrophages (GAMs) (4, 5). Their functions are diverse, including tumor clearance by phagocytosis, antigen presentation, inflammation, blood vessel formation, lipid metabolism, energy production, response to low oxygen, and tissue remodeling, as well as roles in immune regulation, suppression, tumor growth, and metastasis (5, 6). GAMs have broadly been implicated in gliomagenesis by either supporting tumor growth or impeding anti-glioma immunity. Therefore, several preclinical and clinical investigations have pursued pan-GAMs depletion strategies to dissect their incriminating roles and relieve immunosuppressive pressures in the TME of GBM. Since colony stimulating factor-1 receptor (CSF1R) regulates differentiation and maintenance of tumor-associated macrophages (TAMs), therapeutically CSF1R inhibitors have been used to evaluate its role in glioma inhibition. However, incongruent outcomes have been reported, wherein inhibiting CSF1R signaling in GAMs improved survival in GBM mouse models (7); conversely, in a follow-up murine investigation, pan-GAM depletion with CSF1R inhibitors did not render survival benefits in spontaneous experimental GBM models of neural stem cell and oligodendrocyte origins (8, 9). Moreover, CSF1R inhibition in recurrent GBM Phases I and II clinical trials failed to recapitulate therapeutic efficacy observed in preclinical studies (7, 10), thereby raising concerns with such broad targeting approaches, given the coexistence of diverse subpopulations of GAMs with distinct and opposing functions (6, 11).

Immunosuppressive manifestations in glioma TME are exacerbated by the clinical use of dexamethasone, making it challenging to separate its effects from intrinsic myeloid immunoregulatory programs (12). Concurrent with these tumor-supportive programs, IL-10^+^ HMOX1^+^ myeloid cells and *MARCO*, *GPNMB*, *SIGLEC9*, and *SELENOP* subsets of MACs in human studies have been described to negatively regulate T-cell functions in GBM (13–16). Conversely, pro-phagocytic CD169^+^ MACs, TREM2^+^ MG subsets, cytotoxic myeloperoxidase^+^ MACs, and granulysin^+^ MG subpopulations exemplify notable anti-tumor GAMs identified in recent single-cell human glioma studies, indicating functions beyond largely ascribed immunosuppressive behaviors (2, 17–19). Together, these findings highlight the functional diversity of GAMs, underscoring the need to move beyond the conventional view of GAMs being uniformly immunosuppressive and to better characterize the context-dependent roles of distinct myeloid subsets in GBM.

Since MACs can arrest tumor growth, herein, we utilized our glioma immune atlas datasets (2) and performed ligand-receptor (L-R) interactome analyses to gain insights into anti-glioma effectors of GAMs. Our systematic analyses of L-R pairs between glioma-associated myeloid and lymphoid lineage cells identified *LGALS9* (encoding Galectin-9)*/HAVCR2* (Tim-3) as one of the predominant interactions, which has been attributed to regulating T-cell death and is a pursued target for cancer immunotherapy (20). Beyond the trans-Galectin-9/Tim-3 inhibitory functions in the context of exhausted T and NK cells, independent functions of Tim-3 in negatively regulating microglial phagocytosis have been reported, but the endogenous function of Galectin-9 remains unexplored (21). Therefore, in this study, we set out to understand the expression patterns of Galectin-9 in immune and tumor cells of human glioma TME and their functional implications in GAMs. Altogether, our interrogation of the glioma microenvironment provides insights into the interactome diversity of myeloid and lymphoid cells across isocitrate dehydrogenase (IDH) wild-type (IDH-wt) and IDH-mutant (IDH-mut) gliomas. Specifically, we highlight the unappreciated non-canonical role of Galectin-9 in MG as regulator of glioma cell adhesion and phagocytosis that can be harnessed for GBM immunotherapy.

## 2 Materials and methods

### 2.1 Collection of human brain tumor and tissue (henceforth termed as “tissue”)

The brain tissues from 56 glioma patients undergoing neurosurgery after informed consent as per the internal review board (IRB) approved protocol numbers—LAB03-0687, LAB04-0001, and 2012-0441 [detailed clinical characteristics described in the Gupta and Minghao et al. study (2)]—were collected at The University of Texas MD Anderson Cancer Center (MDACC). Five quasi-normal epileptic (non-glioma brain, NGB) tissues were procured from patients undergoing neurosurgery as per IRB-approved protocol number H-13798 at Baylor College of Medicine. All experiments were compliant with the IRB of MDACC, USA. For primary human microglia (pMG) cultures, tissue collection protocols were approved by the Albert Einstein College of Medicine, Rutgers University, and the University of Texas Medical Branch IRB approved Protocol Numbers Pro20140000794, Pro2012001303, 18-0136, 18-0135, and 18-0134.

### 2.2 Single-cell RNA sequencing (sc-RNAseq)–based Ligand-receptor (L-R) interactome analysis

To identify significant L-R pairs between myeloid and lymphoid cells, we used a dataset of MG, DC, CD4^+^ T, Treg, CD8^+^ T, NK, and NKT defined in sc-RNAseq analyses as previously reported (2). Then, we defined the highly expressed genes as per the rule that more than 20% of cells had its expression level log2(normalized UMI + 1) > 0.5 in a certain cell type. Thereafter, we matched these highly expressed genes with the experimentally validated published L-R list (22) to identify the potential ligands and receptors. We assume two cell types have interaction when one highly expresses a ligand/receptor and the other one highly expresses the paired receptor/ligand. For the differential interactome analysis, we found the differentially expressed genes between IDH-wt and IDH-mut using the function *FindMarkers* from *Seurat* with parameter min. pct = 0.2 and then matched these differentially enriched genes (DEGs) with the ligand-receptor database based on (L-R) co-expression using CellPhoneDB v2.0 (23). We defined increased interactions as when the expression of an L-R pair was upregulated while its pair was not downregulated in one group.

### 2.3 Spectral cytometry dataset

As per our previously published 40-plex cytometry dataset (2), we evaluated the percentage of PD-1, PD-L1, Galectin-9, and Tim-3 expressing MG, MACs, monocyte-derived macrophages (MDM), cDC1, and cDC2 across glioma subtypes: IDH-mut primary (IMP, *n* = 14), IDH-mut recurrent (IMR, *n* = 9), IDH-wt primary (IWP, *n* = 13), IDH-wt recurrent (IWR, *n* = 12), and NGB (*n* = 3).

### 2.4 Fluorescence-activated cell sorting of tumor and associated leukocytes

Single-cell suspensions of tumor obtained from three GBM patients were isolated and cryopreserved as previously described (2). Cryopreserved cells were thawed and washed with complete 10% fetal bovine serum (FBS) in RPMI 1640 (10-040-CV; Corning, Manassas, VA, United States), 1% penicillin-streptomycin (P4333; Sigma-Aldrich), and 10 mM 2-[4-(2-hydroxyethyl) piperazin-1-yl] ethanesulfonic acid (R5158; Sigma-Aldrich) and incubated at 37°C for 30 min. Further, the cells were washed with 2% FBS. Next, cells were incubated with FcR Blocking Reagent (130-059-901; Miltenyi Biotec, 1:20 dilution) for 10 min at RT. Thereafter, cells were incubated in the dark with a 1:200 dilution of anti-human CD45-APC (130-113-114; Miltenyi Biotec) for 20 min at 4°C. Tumor cells (CD45^-^) and leukocyte (CD45^+^) cells were sorted by live/dead Sytox-Green staining (S7020; Thermo Fisher Scientific) using a BD FACS Aria III (Becton Dickinson).

### 2.5 Western blotting

Total cell protein was extracted from flow-sorted tumor, leukocytes of GBM patients (CA28, CA29 and CA158), and GSC-23 and GSC-28 were extracted by RIPA buffer containing protease inhibitor and phosphatase inhibitor (TargetMol). Protein was quantified and separated by SDS-PAGE. Primary antibodies were diluted and incubated overnight at 4 °C. The membranes were then washed with TBST and incubated with HRP-conjugated secondary antibodies (1:3000 dilution) (GenDEPOT) at room temperature for 1h. Enhanced chemiluminescence (GenDEPOT) was performed. Anti-Galectin 9 antibody (Cat# 54330, 1:1000 dilution) and anti-β-actin antibody (Cat# A5441, 1:10000 dilution) were purchased from Cell Signaling Technology and Millipore Sigma, respectively.

### 2.6 Multiplex immunohistochemistry (IHC)

5μm sections of formalin-fixed paraffin-embedded (FFPE) brain tissue of glioma patients were deparaffinized in Xylene (3 min, 2 times), dehydrated in 1:1 (v/v) Xylene: 100% ethanol for 3 min, and washed with 95%, 70%, and 50% ethanol for 3 min. Tissue sections were rehydrated with a dH_2_O wash (3 min). Antigen retrieval was performed with 10 mM Tris/EDTA Buffer (pH 9.0). Microwave slides for 10 min at 100% power and cool at RT for 30 min, followed by 3× washes with dH_2_O for 1 min each. Tissue autofluorescence blocking steps included True Black (Biotum, 23007 diluted in 70% ethanol) for 5 min and Block BF in 1:10 FcR blocker and Background Buster (NB309+NB309) for 15 min with 3× dH2O washes of 1 min after each step. FFPE sections were stained overnight with primary antibody chicken anti-Iba1 (1:1000 dilution, Synaptic Systems 234 009), rabbit anti–Galectin-9 (1:500 dilution, CST 54330), and mouse anti-Nestin (1:500 dilution, Sigma MAB5326) at RT. The excess of primary Ab were washed for 1 min in ASB and 3× 1 min in dH2O. Secondary staining with DAPI (1:10000), 1:500 goat anti-chicken Alexa Fluor 594, 1:500 goat anti-rabbit Alexa Fluor 488 (Invitrogen A11034), and 1:500 goat anti-mouse IgG2b-Alexa Fluor 546 (Invitrogen A11034) for 120 min at RT was performed, and washed for 1 min in ASB and 3× 1 min in dH2O. Air-dry sections for 15 min, cover with Immu-Mount (Fisher, 99-904-02) and seal with 22 × 50 mm coverslip. Slides were viewed in Keyence BZ-X800.

### 2.7 Differential gene enrichment analyses of Galectin-9^hi/+^ versus Galectin-9^lo/−^ MG, MAC/MDM

MG and MACs (MAC + MDM) cell types from the sc-RNAseq dataset of IDH-wt as previously reported (2) were extracted as a starting point for this analysis. Quantile expression (Q ≤ 0.25) of *LGALS9* gene was defined as Galectin-9^lo/−^ (hereafter referred to as Galectin-9^−^) and Q ≥ 0.75 as Galectin-9^hi/+^ (hereafter referred to as Galectin-9^+^) in respective cell types. Following this, the *RunUMAP* function was used to see if the clusters are well dispersed. An *EnhancedVolcano* (version 1.20.0) plot was generated using a *p-adjusted* value cutoff of 0.05 and *avg. log2FC* of 0.5 to highlight the top variable genes. A list of phagocytic markers was compiled from the Gene ontology webserver (24) and the enrichment of these markers in the Galectin-9 subpopulations was obtained using an *adjusted* value cutoff of 0.05. The union set of the differentially enriched phagocytic markers was visualized with a *DotPlot* (Seurat version 5.3.0).

### 2.8 Culturing of human pMG

Cultures of pMG from neocortex and occipital areas were prepared as described for human fetal microglia (25). Tissue was minced and incubated at 37 °C for 30 min in Ca^2+^-free phosphate buffered saline (PBS) that contained trypsin (0.5%) and EDTA (5 mM). After removal of the enzyme solution, tissue was triturated in dissociation medium (MEM medium, 1 mg/ml bovine pancreas DNAase I, and 10% horse serum) using a Pasteur pipette. Dissociated cells were pelleted, resuspended in MEM medium supplemented with 10% FBS, 100 units/ml penicillin, and 50 mg/ml streptomycin sulfate, and plated on glass coverslips or on 60 mm plastic culture dishes (Nunc, Roskilde, Denmark) and kept at 37°C in a 5% CO_2_/95% air atmosphere at nearly 100% relative humidity. Cells were fed every three days. About 95% of the cells were GFAP-positive, indicating that cultures were highly enriched in astrocytes; presumably most of the remaining 5% were pMG. Because pMG proliferates in astrocyte-conditioned medium, feeding of confluent cultures was stopped for the following 40–60 days. Cultures were then rigorously agitated for 30 min in an orbital shaker (Lab-Line Instruments, Inc.) at 150 rpm and 37 °C to detach nonadherent cells from the astrocyte monolayer. Thereafter, pMG suspended in the culture medium were collected and plated (800,000 cells/ml; 3 ml on 60 mm dishes of 1 ml per well or a 24-well multiplate (Nunclon, Roskilde, Denmark), each containing a round glass coverslip at the bottom or Ibidi culture chambers inserts—two wells (Ibidi, catalog, 81176). After 15–30 min, nonadherent cells were discardedm and adherent cells were maintained in DMEM medium supplemented with 10% fetal bovine serum, 100 units/ml penicillin, and 50 mg/ml streptomycin sulfate. Close to 99% of the cells obtained after this procedure were immunopositive for Iba-1 and negative for GFAP, indicating a very high enrichment in pMG. All experiments were performed 48–72h after subculturing pMG at 37 °C in a 5% CO_2_/95% air atmosphere at nearly 100% relative humidity (see coculture methods).

### 2.9 Live cell imaging

For analysis, two different cell culture systems were used. First, mixed cultures in mattek plates. Also, Ibidi two-chamber cultures were used with GFP and pMG cells in each chamber. Upon siRNA or antibody treatment, the barrier was removed to enable interactions between both cell types. Our imaging system corresponds to an Axio-observed Z1 with three redundant incubation systems with CO_2_ and humidity control, as well as a confocal A1R with a full incubation system and live cell imaging. We imaged up 2h with recording every 1 min.

### 2.10 pMG and GSC co-culture models

pMG and GSC8-11ZsG cells were cultured in Ibidi plates with a silicone ring separating both cell types. To assess phagocytosis, the silicone ring was removed that allowed interaction between both cell types. Thereafter, adhesion and phagocytosis were assessed by live cell imaging analyses as previously described (26). Phagocytosis and adhesion ratio were calculated as the number of pMG that phagocytosed or adhered with GSC8-11ZsG divided by total number of pMG with or without phagocytosis or adhesion events in at least 10–15 different fields of view.

### 2.11 Immunofluorescence staining

GFAP and pMG cells were fixed and permeabilized in 70% ethanol for 20 min at −20°C. Cells were incubated in blocking solution for 30 min at room temperature and then in primary antibody (anti-Iba-1 or Galectin-9 0: at 1:500 dilution) for 2h at room temperature. Cells were washed several times with PBS at room temperature and incubated with the appropriate secondary antibody conjugated to FITC or Cy3 (Sigma, St. Louis, MO) for 1h at room temperature, followed by another wash in PBS for 1 h. Coverslips were then mounted using antifade reagent with DAPI. Cells were examined using an A1 Nikon confocal microscope with spectral detection, and image analyses were performed using NIS element software (Tokyo, Japan).

### 2.12 Image analysis

Raw data for cell–cell interactions were obtained in NIS elements (Nikon, Japan). For confocal analysis, 3D deconvolutions were obtained using NIS elements (Nikon, Japan). Quantification of colocalization, intensities, and lengths as well as stability was performed in NIS elements and ImageJ.

### 2.13 Statistical analyses

For cytometry data, statistical differences were determined using Kruskal–Wallis test followed by Dunn’s post-hoc test for multiple comparisons at indicated *p*-values between NGB versus glioma subtypes, IMP versus IMR, IWP versus IWR, and IMP versus IWP. GraphPad Prism v. 10.5.0 was used for data analysis. For pMG-GSC co-culture assays for cell adhesion and phagocytosis, statistical differences were determined using the Kruskal–Wallis test followed by Dunn’s post-hoc test for multiple comparisons between siRNA-treated versus control groups and Galectin-9 Ab-treated versus control groups at *p < 0.05, **p < 0.01, ****p < 0.001, and ****p < 0.0001.

## 3 Results

### 3.1 L-R interactome analyses reveals Galectin-9/Tim-3 as a major checkpoint axis in gliomas

**Figure 1:**
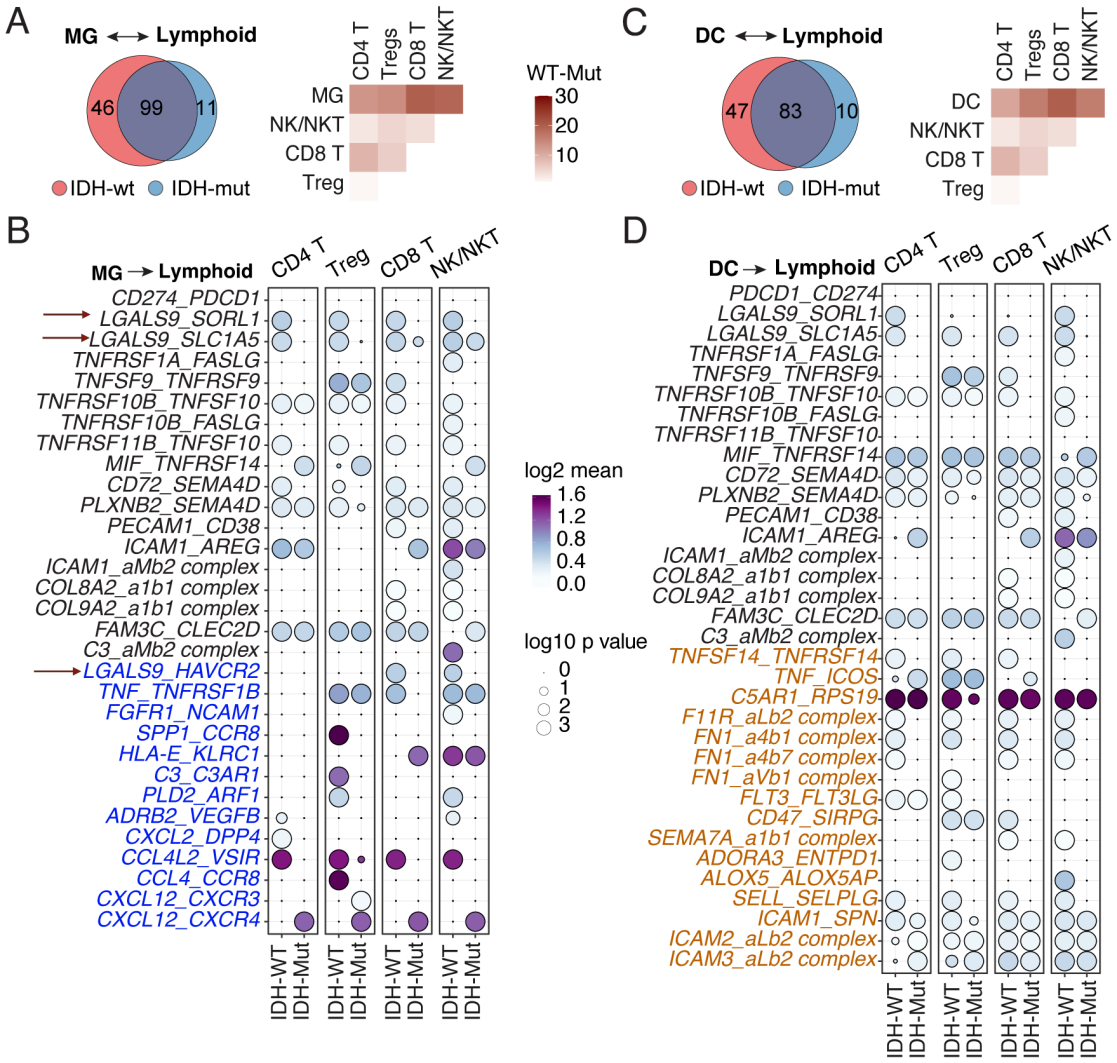
L-R–based cellular interactions between glioma-associated MG, DCs, and lymphoid cell types. **(A, C)** Venn diagrams show the number of overlapping and unique L-R–based interactions in IDH-wt and IDH-mut gliomas. Corresponding, heatmaps show the number of interactions between MG in **(A)** and DCs in **(C)** with indicated leukocytic subpopulations as inferred by CellphoneDB between IDH-wt versus IDH-mut gliomas. Scale depicts the number of gained interactions in IDH-wt gliomas relative to the IDH-mut subtype. **(B, D)** Bubble plots showing the mean expression (color key) and significance (size key) of L-R pairs between indicated lymphoid cell types and MG in **(B)** and DC in **(D)**. Only interactions that showed differences between IDH-wt and IDH-mut are shown along with differential MG-associated (colored blue) and DC-associated (colored orange) interactions. Highlighted L-R interactions (dashed red) in **(B)** shows pertinent *LGALS9* interacting receptors, of which *LGALS9-HAVCR2* were characterized for their protein expression with spectral cytometry in relevant cell types.

Coordinated cellular communication between innate and adaptive immune cells is essential for effective responses against tumors. In this context, sc-RNAseq–based L-R interactions have been investigated between myeloid cells and tumors to identify communication networks and therapeutic targets in GBM (27, 28), but to a lesser extent between MG, DCs, and lymphocytes. To identify these interaction networks, we utilized immune cell types defined in our pan-glioma sc-RNAseq atlas, which includes primary and recurrent IDH-wt (*n* = 8) and IDH-mut (*n* = 10) patients (clinical characteristics previously described in (2)). We inferred interactions between different myeloid and lymphoid cells based on L-R co-expression using CellPhoneDB v2.0 (23). The L-R gene list used for identifying the glioma-associated interactome was derived from an experimentally validated resource (29). L-R pairs were directionally concatenated from phagocytic sentinels (MG and DC) to lymphoid (CD4^+^ T, Treg, CD8^+^ T, NK, and NKT) cells (**Figures 1A–D**). Although most of the immune interactions were shared between IDH-wt and IDH-mut, some private interactions were seen for each glioma subtype, of which IDH-wt displayed a higher number of unique interactions in both MG and DCs (**Figures 1A, C**). Overall, among the significant interactions, TNF family, comprising both co-stimulatory (*TNFRSF9*, *TNFRSF14*, and *ICOS*) and co-inhibitory (*VSIR*) were overrepresented L-R interaction amongst both MG and DC with the indicated cell types (**Figures 1B, D**). In addition, we observed MG-specific (chemokine family) and DC-specific (adhesion molecules, *ICAM* and *PECAM*, and fibronectin-integrin family) interactomes (**Figures 1B, D**). Notably, our interactome analysis revealed three nodes of *LGALS9* encoding Galectin-9 interactions, such as *LGALS9-SORL1* and *LGALS9-SLC1A5* (**Figures 1B, D**), of which *HAVCR2* encoding Tim-3 has been well characterized as a co-inhibitory axis in other malignancies (30), but sparingly studied in glioma beyond their role in T-cell dysfunction and macrophage polarization (31). Compared to the highly studied PD-1/PD-L1 inhibitory interaction, our results suggest Galectin-9/Tim-3 as the dominant axis between MG and CD8^+^ T and NK/NKT cells in IDH-wt gliomas (**Figure 1B**). In GBM, *CXCL12-CXCR4* interactions are pathologically associated with the proliferation of malignant cells (32). Intriguingly, we observed MG exhibited increased *CXCL12-CXCR4* interactions with lymphoid lineage cells in IDH-mut rather than IDH-wt gliomas (**Figure 1B**). In summary, our interactome analysis sculpts the comparative communication map of individual phagocytes with different lymphoid lineage cells harbored in the TME of IDH-mut and IDH-wt human gliomas.

### 3.2 Galectin-9 protein is expressed in glioma associated myeloid cells across glioma subtypes

**Figure 2:**
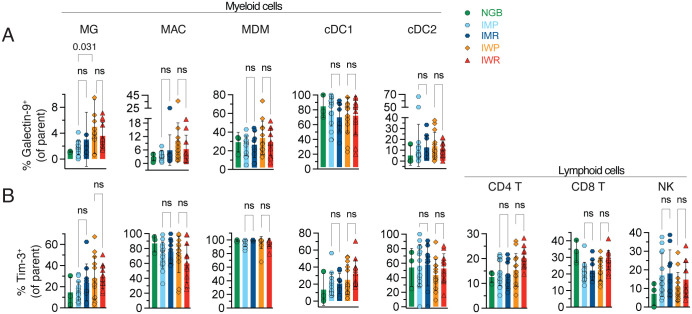
Galectin-9 and Tim-3 expression assessed by cytometry across glioma subtypes. **(A, B)** Color-coded scatter bar plots represent the relative proportions of Galectin-9^+^ in A, Tim-3^+^ in B cells of indicated myeloid and lymphoid cell types across IDH classified primary and recurrent gliomas: NGB (*n* = 3), IMP (*n* = 14), IMR (*n* = 9), IWP (*n* = 13), IWR (*n* = 12). Statistical differences were determined using Kruskal–Wallis test followed by Dunn’s post-hoc test for multiple comparisons at indicated p values, between NGB versus glioma subtypes, IMP versus IMR, IWP versus IWR, and IMP versus IWP. n.s. = statistically not significant. See also **Supplementary Figure S1**.

Because inhibiting Galectin-9/Tim-3 axis suppresses glioma growth in experimental GBM mouse models (33, 34), and this axis remains insufficiently characterized beyond GBM infiltrating T cells (35), it becomes imperative to dissect their expression patterns in both IDH-mut and IDH-wt gliomas for translational relevance. Hence, we immunoprobed Galectin-9 and Tim-3 in phagocytes (e.g., MG, MACs, MDM, DCs) across the TME of the primary and recurrent IDH-wt (n = 12-13) and IDH-mut (*n* = 9–14) glioma cohort using a previously reported 40-plex spectral cytometry panel (2). Our analyses corroborated the dominance of Galectin-9/Tim-3 expression across myeloid lineage phagocytes (**Figures 2A, B**) comparative to PD-L1/PD-1 axis in gliomas (**Supplementary Figures S1A, C**). Given MG constitutes 20%–70% of leukocytes in glioma TME (2), even though Galectin-9^+^MG comprised 2%–8% of the total MG in IDH-wt gliomas, these cells were markedly abundant and significantly higher in IDH-wt primary (IWP) compared to IDH-mutant primary (IMP) gliomas (**Figure 2A**). As expected, PD-L1 expression on MG was noted at protein level but not their cognate receptor PD-1 (**Supplementary Figure S1C**). We observed significantly increased proportions of PD-L1^+^MG in IDH-wt recurrent (IWR) compared to IWP glioma subtype (**Supplementary Figure S1C**). Tim-3^+^ CD4^+^ and CD8^+^ T-cell subsets were higher than PD-1^+^ subpopulations but were comparable across gliomas (**Figure 2B**, **Supplementary Figure S1D**). Our results support previous findings (36, 37) that tumor infiltrating Tim-3^+^CD4^+^ and Tim-3^+^CD8^+^ T cells in primary and recurrent GBM are quantitatively comparable, which we observed is even true for primary and recurrent IDH-mut gliomas (**Figure 2B**). Moreover, frequencies of Tim-3^+^PD-1^+^ CD4 T and CD8 T lymphocytes were higher in IDH-wt gliomas (**Supplementary Figure S1F**). As previously reported, PD-L1 can be expressed by tumor-associated NK cells (38). Unlike CD8 T cells, proportions of Tim-3^+^, PD-1^+^, PD-L1^+^, and PD-1^+^Tim-3^+^ NK subpopulations were not differential between the glioma subtypes (**Figures 2A, B**, **Supplementary Figures S1B, D, F**). These results thoroughly describe the comparative expression patterns for Galectin-9/Tim-3 and PD-L1/PD-1 across glioma-associated myeloid and lymphoid lineage cells. Intriguingly, we identify Galectin-9-expressing MG present in IDH-wt gliomas, worthy of further investigation.

### 3.3 Malignant glioma cells do not express Galectin-9 protein

**Figure 3:**
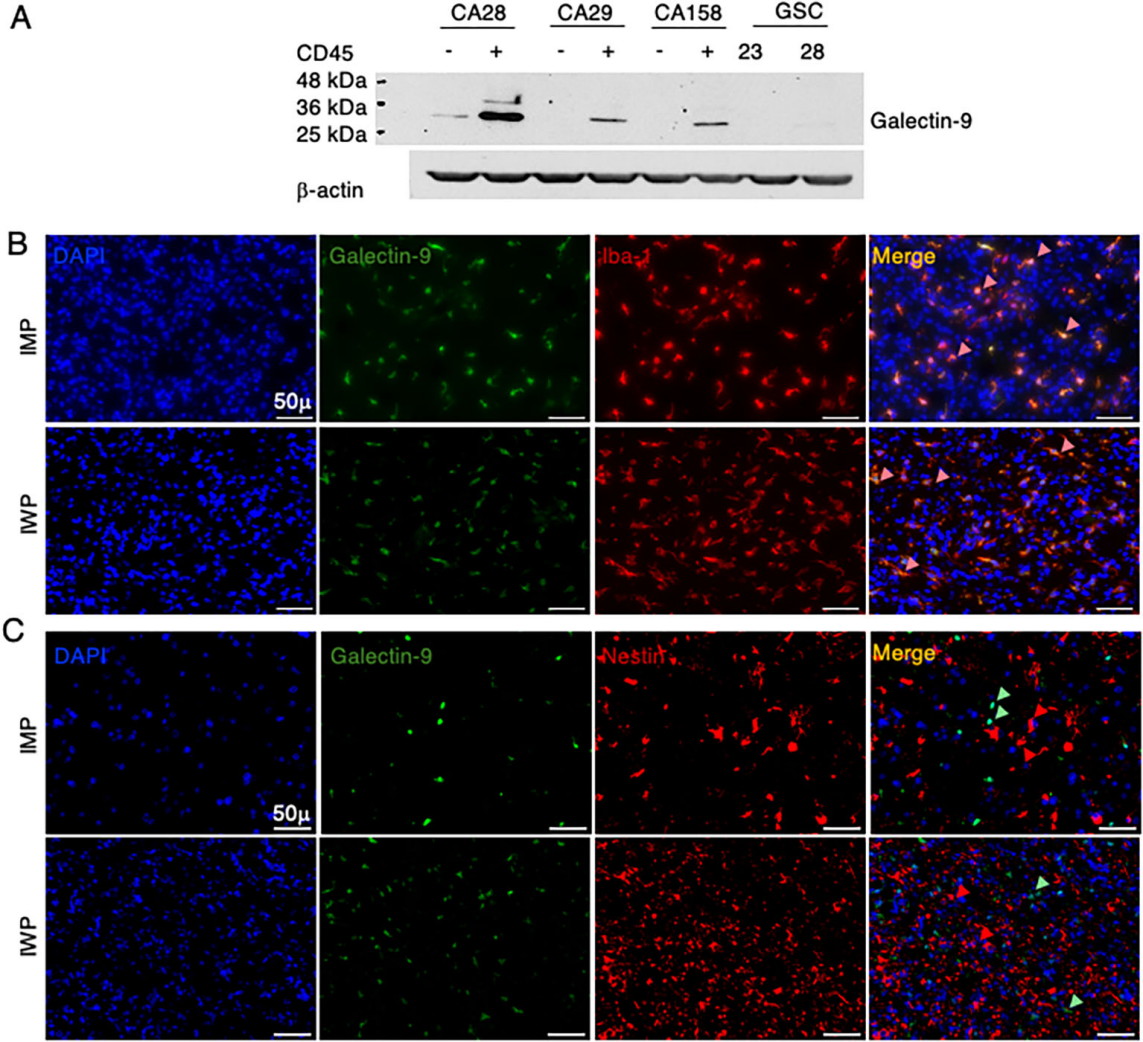
Galectin-9 expression in tumor and associated leukocytes in glioma. **(A)** Western blot showing expression of Galectin-9 in flow-sorted CD45^-^ (tumor), associated leukocytes (CD45^+^), GSC-23, and GSC-28. **(B)** Representative microscopic image of multiplex IHC stained FFPE tissue sections of primary IDH-mut (top) and IDH-wt (bottom) glioma patients (*n* = 5/group). Unmixed images showing expression of Galectin-9 (green), Iba-1 (red), and DAPI (4”,6-diamidino-2-phenylindole) and composite image showing co-expression of Galectin-9^+^Iba-1^+^ MG (crimson, highlighted by arrows) at 40× magnification. Scale bars = 50 μm. **(C)** Representative microscopic image of multiplex IHC stained FFPE tissue sections of primary IDH-mut (top) and IDH-wt (bottom) glioma patients (*n* = 5 per group). Unmixed and composite images showing expression of Galectin-9 (green), Nestin (red), and DAPI (4”,6-diamidino-2-phenylindole) at 40X magnification. Nestin expressing glioma cells do not express Galectin-9 as shown by red and green arrows. Scale bars = 50 μm.

Transcriptomic correlation studies have implicated Galectin-9 expression with poor survival of mesenchymal GBM (39, 40); however, whether Galectin-9 is expressed by malignant cells in human glioma TME needs to be determined. Therefore, we characterized the expression of Galectin-9 protein using flow-sorted GBM-associated leukocytes (CD45^+^) and malignant cells (CD45^−^) from three different GBM patients and two different GSC lines (GSC-23 and GSC-28) by Western blot assessments. Our observations confirm expression of total Galectin-9 protein (membrane and cytosolic) in glioma-associated leukocytes and not in malignant cells (**Figure 3A**). Using the orthogonal immunofluorescence approach, we corroborated the expression of total Galectin-9 in Iba-1^+^ phagocytes and not in Nestin^+^ malignant cells across five IWP and IMP glioma patients (**Figures 3B, C**). These results substantiate that Galectin-9 is not expressed by glioma cells but mostly restricted to myeloid cells.

### 3.4 Adhesion and phagocytosis-associated genes are enriched in Galectin-9^+^ MG and MACs

**Figure 4:**
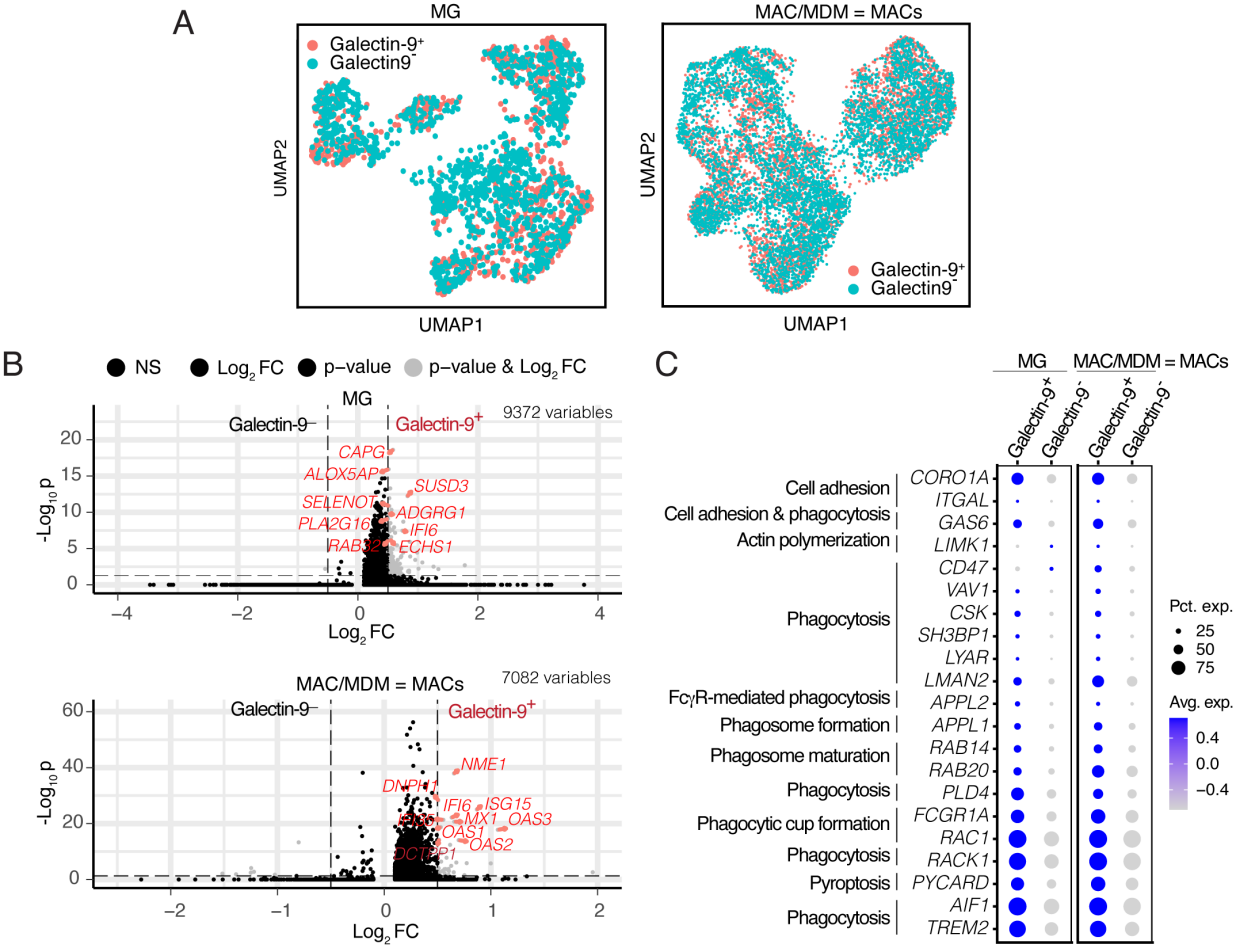
Gene enrichment analysis of Galectin-9^+^ and Galectin-9^-^ subpopulations of GAMs. **(A)** UMAP visualization of MG (left), MAC/MDM = MACs (right) based on differential expression of Galectin-9 gene in IDH-wt glioma patients (*n* = 8). Cells are color coded for Galectin-9 expression. **(B)** Enhanced volcano plot of the variable genes in (*n* = 9,372) (top) and Galectin-9^+^ MACs (bottom) compared to Galectin-9^-^ counterparts. Gray dots represent genes qualifying average log2FC cutoff of 0.5 and adjusted *p*-value cutoff of 0.05. The top significant genes for Galectin-9^+^ GAMs indicated in red. **(C)** Bubble plot representing the union set of phagocytic markers differentially enriched in Galectin-9^+^ and Galectin-9^-^ subpopulations of MG and MACs as indicated. The genes are annotated for their molecular function. The scaled gene expression is shown by the color-bar and the percentage expression by cells is represented by dot size.

Due to the abundance of Galectin-9^+^MG in IDH-wt gliomas, we extracted MG and MAC/MDM cells (collectively referred to as MACs) from our sc-RNAseq IDH-wt cohort (2), and surprisingly, Galectin-9-specific clusters of MG and MACs were not observed rather its expression was cluster agnostic (**Figure 4A**). Therefore, we stratified them based on differential Galectin-9 expression to identify associated enriched immunological pathways (**Figure 4A**). Next, we performed differential gene enrichment analyses on Galectin-9^+^ versus Galectin-9^−^ subpopulations. Amongst the 9,372 gene variables in MG, we identified Sushi domain containing 3 (*SUSD3*), Selenoprotein T (*SELENOT*), and macrophage capping protein *(CAPG)*, and a G-protein coupled receptor *(ADGRG1/GPR56)* as top cell adhesion (41, 42) and phagocytosis-associated (43, 44) genes, respectively, in Galectin-9^+^MG compared to Galectin-9^-^MG subsets (**Figure 4B**). Even Galectin-9^+^ MACs showed enrichment of metastatic suppressor gene *(NME1)*, known to inhibit cell proliferation by phagocytosis (**Figure 4B**). Upon deeper investigation into genes associated with cell adhesion (e.g., *CORO1A* encoding coronin 1A, *ITGAL* encoding integrins), phagosome formation (*APPL1, RAB14, RAB20*), phagocytic cup formation *(FCGR1A, RAC1)*, and phagocytosis *(TREM2, AIF1)* (19, 24), we observed genes associated with these biological pathways were enriched in Galectin-9^+^ subpopulations regardless of MG and MACs origin. It became increasingly clear that Galectin-9 potentially plays a putative role in cellular adhesion and phagocytosis processes (**Figure 4C**).

### 3.5 Galectin-9 expression in microglia mediates glioma cell adhesion

**Figure 5:**
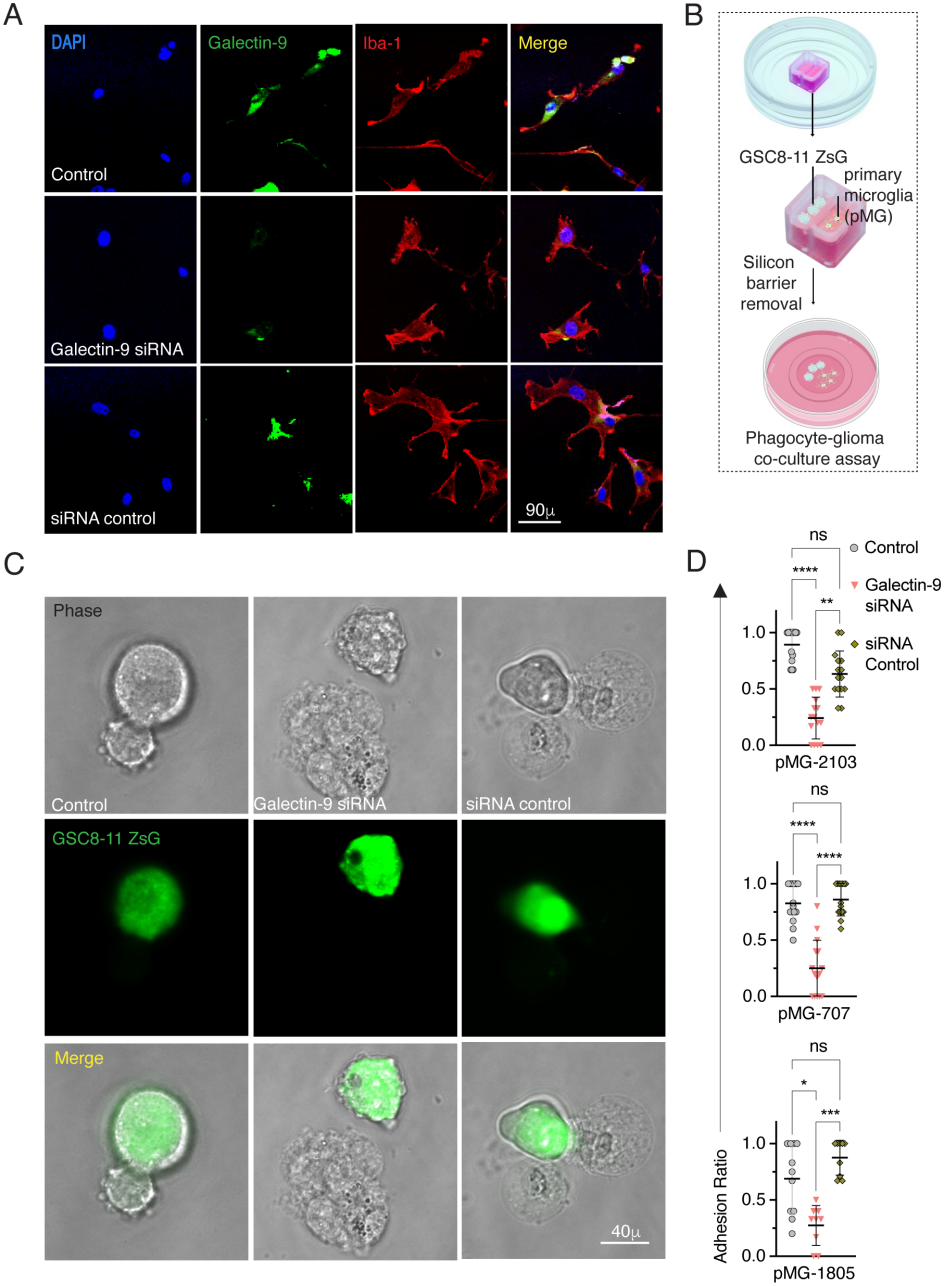
Glioma cell adhesion was reduced upon Galectin-9 downregulation. **(A)** Representative microscopic immunofluorescence image showing staining of Galectin-9 (green), Iba-1 (red) and DAPI and their composite expression in merged image of untreated pMG controls (top), Galectin-9 siRNA treated pMG (middle) and siRNA controls (bottom). Scale bars = 90 μm. **(B)** Diagram showing Ibidi experimental design for pMG/GSC8-11ZsG co-culture assays for adhesion and phagocytosis. **(C)** Representative microscopic immunofluorescence image showing phase contrast visualization of adhered pMG and GSC8-11ZsG (green) calculated as adhesion ratio when cocultured with untreated pMG controls (top), Galectin-9 siRNA–treated pMG (middle), and siRNA controls (bottom) with GSC8-11ZsG at 2h post-incubation. Scale bars = 40 μm. **(D)** Scatter dot plots showing corresponding proportions as mean ± SD of % GSC8-11ZsG adhered to indicated pMG from three different fetal donors (pMG-2103, pMG-707, and pMG-1805). Error bars indicate the SD of mean. Statistical differences were determined using Kruskal–Wallis test followed by Dunn’s post-hoc test for multiple comparisons between siRNA treated versus control groups at *p < 0.05, **p < 0.01, ***p < 0.001, and ****p < 0.0001. See also **Supplementary Figures S2** and **S3**.

Determining whether Galectin deficiency–associated phenotypes are driven by intracellular or extracellular pathways remains a significant *in vivo* challenge (45). In addition, experimental murine GBM immunopathogenesis is not comparable to human gliomas (46); therefore, we generated primary microglial cultures (pMG) as previously described (25) from three different human subjects annotated as pMG707, pMG2103, and pMG1805. Phase contrast and immunofluorescence microscopy confirmed the MG-like ramified morphology and co-localization of Galectin-9 with Iba-1, indicating pertinent Galectin-9 expression across all naïve pMG cultures (**Supplementary Figure S2**). These pMG were amenable to siRNA knockdown. Resultantly, Galectin-9 expression was effectively downregulated in Galectin-9 siRNA-treated MG cultures compared to siRNA- and untreated controls (**Figure 5A**). To evaluate the functional role of Galectin-9 on MG in glioma microenvironment, MG *ex-vivo* coculture system was developed that involved seeding of pMG with GSC8–11 expressing ZsG reporter (GSC8-11ZsG); a patient-derived glioma stem cell line (**Figure 5B**). Next, we tested the effect of Galectin-9 silencing on pMG behavior. Intriguingly, confocal immunofluorescence imaging of co-cultures revealed that silencing of Galectin-9 gene on pMG significantly reduced their adhesion to GSC8-11ZsG compared to untreated and siRNA-controls across all three pMG (**Figures 5C, D**). As Galectin-9 can manifest these observed phenotypes due to intracellular or extracellular activity or both, and that siRNA can cause downregulation of total gene product, it can be inferred that attenuated adhesive properties in Galectin-9 silenced pMG resulted from decrease in total Galectin-9 protein. To test whether neutralizing surface Galectin-9 would phenocopy reduced adhesion phenotype in pMG, we utilized MAb-13; a Galectin-9 neutralizing monoclonal antibody that has been shown to effectively neutralize Galectin-9 (47). MAb-13 mediated neutralization of Galectin-9 in MG707 significantly reduced the adhesion of pMG cells with GSC8-11ZsG relative to untreated and IgG controls, strongly suggesting a functional consequence of extracellular Galectin-9 receptor on adhesion properties of pMG (**Supplementary Figure S3A**).

### 3.6 Galectin-9 regulates microglial phagocytosis of glioma cells

**Figure 6:**
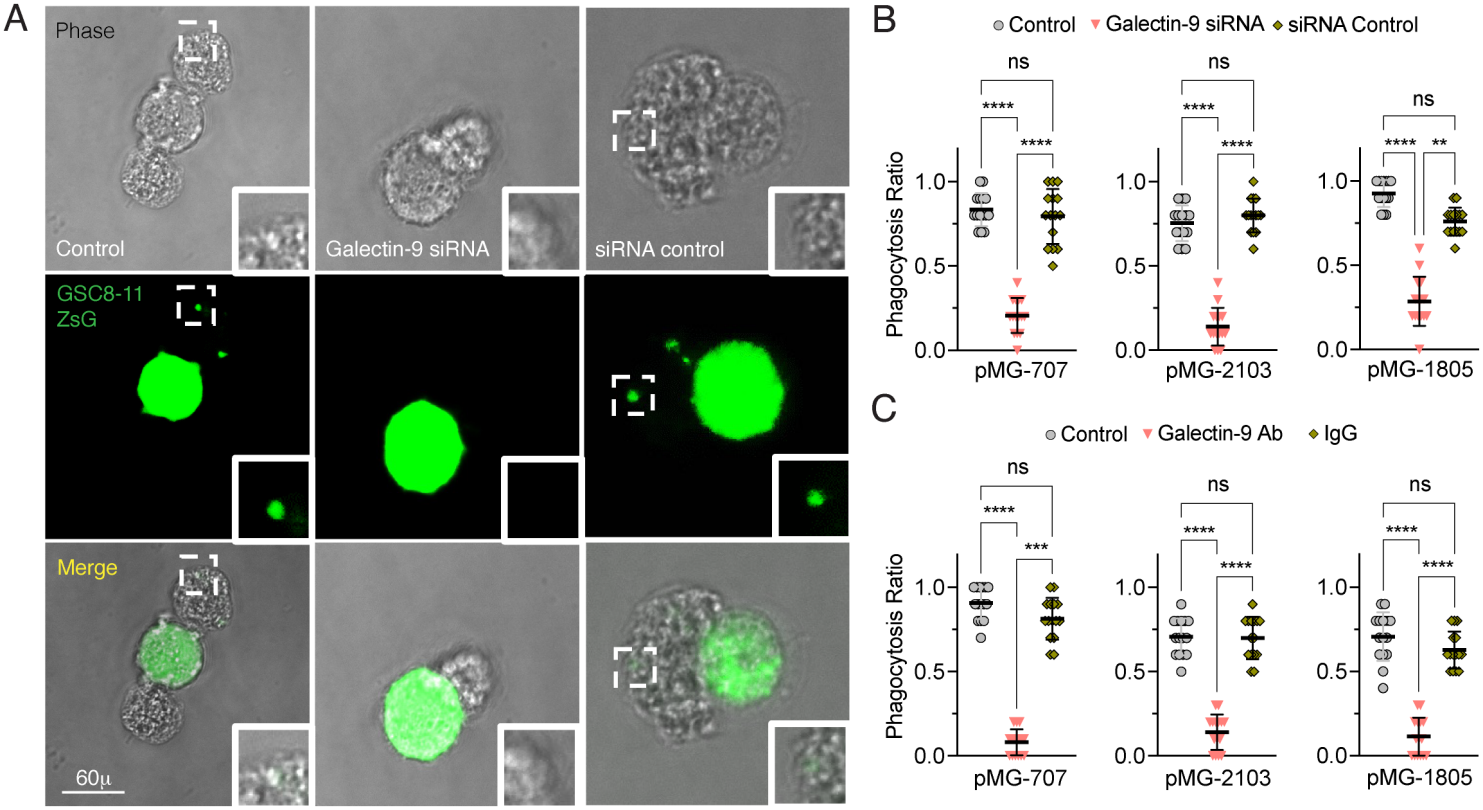
Impaired phagocytic uptake of glioma cell by pMG upon Galectin-9 downregulation. **(A)** Representative microscopic immunofluorescence image showing phase contrast visualization of pMG, GSC8-11ZsG (green) and pMG/GSC8-11ZsG (merged) exhibiting phagocytosis when cocultured with untreated pMG controls (top), Galectin-9 siRNA–treated pMG (middle) and siRNA controls (bottom) with GSC8-11ZsG at 2h post-incubation. Scale bars = 60 μm. White boxes shows magnified image that depicts amount of GSC8-11ZsG by pMG. **(B)** Scatter dot plots showing mean proportions ± SD of pMG (pMG-2103, pMG-707, and pMG-1805) that phagocytosed GSC8-11ZsG (phagocytosis ratio) in untreated control, treated Galectin-9 siRNA, and siRNA control experimental conditions when co-cultured with GSC8-11ZsG. Error bars indicate the SD of mean. Statistical differences were determined using Kruskal–Wallis test followed by Dunn’s post-hoc test for multiple comparisons between siRNA-treated versus control groups at **p < 0.01, ****p < 0.0001. **(C)**, Scatter dot plots showing mean proportions ± SD of pMG (pMG-2103, pMG-707, and pMG-1805) that phagocytosed GSC8-11ZsG (phagocytosis ratio) in untreated control, treated Galectin-9 neutralization Ab (MAb-13), and IgG control experimental conditions when co-cultured with GSC8-11ZsG. Error bars indicate the SD of mean. Statistical differences were determined using Kruskal–Wallis test followed by Dunn’s post-hoc test for multiple comparisons between siRNA-treated versus control groups at ***p < 0.001, ****p < 0.0001. See also **Supplementary Figure S3**.

Using our established pMG/GSC8-11ZsG *ex-vivo* coculture system, akin to a previous report (26), we evaluated the phagocytosis of fluorescent GSC8-11ZsG cells by pMG. We quantified the number of pMG with fluorescent vesicles as an index for phagocytosis in addition to observing this process by live cell imaging. We measured the phagocytosis ratio and the amount of phagocytosed glioma measured as co-localized pixels. Galectin-9 siRNA-mediated silencing of pMG significantly reduced not only the phagocytosis ratio but also the amount of glioma uptake in silenced pMG-707, pMG-2103, and pMG-1805 compared to their siRNA control and untreated counterparts as evaluated by live cell confocal microscopy (**Figures 6A, B**, **Supplementary Figure S3B**). These results imply that the observed reduction in phagocytosis in Galectin-9–silenced pMG is a cumulative effect of cytosolic and intracellular proteins. Furthermore, we investigated whether MAb-13-mediated neutralization of surface Galectin-9 protein would result in similarly impaired phagocytosis outcomes. Indeed, MAb-13–treated pMG exhibited significantly impaired phagocytosis ratio and reduced quantities of phagocytosed glioma cells relative to untreated and IgG controls (**Figure 6C** and **Supplementary Figure S3C**). These findings indicate functionally redundant MG behaviors independent of Galectin-9 localization.

## 4 Discussion

Through an in-depth single-cell characterization of macrophage diversity in brain TME across primary, relapsed glioma, and metastatic brain tumors, a better understanding of shared and distinct cellular dynamics, heterogeneity, functional plasticity, polarization states, and immunosuppressive programs has emerged (2, 12, 16, 27, 28, 48). Nonetheless, the anti-glioma potential of macrophages remains insufficiently explored. In this study, as an extension to our previous glioma TME interrogations (2), we sought to identify molecular mediators that contribute to the anti-tumor biology of macrophages.

sc-RNAseq-based L-R interactome analyses present a robust, unbiased approach to infer an array of autocrine and paracrine L-R molecules for downstream hypothesis testing (23, 49). Herein, we exemplify an avenue of translational utility of our glioma immune atlas (2), which we utilized to create a shared and differential interactome map between myeloid and lymphoid cells in IDH-wt and IDH-mut gliomas. Overall, we observed a higher number of interactions between MG, DCs, and lymphocytes in IDH-wt gliomas compared to IDH-mut cases, indicating heightened myeloid immune reactivity and crosstalk within IDH-wt ecosystems. In addition to various known co-inhibitory and co-stimulatory interactions, Galectin-9 L-R axis of MG, and DCs engaged multiple receptors (e.g., Sortilin related receptor *SORL1*, glutamine transporter *SLC1A5*) in lymphocytes of IDH-wt glioma with varying degrees. These analyses identify putative myeloid Galectin-9 partners beyond widely studied T cell–associated Tim-3 trans-interactions. While not much is known about the role of these receptors in GAM functions until recently, SorLA has been described to regulate inflammatory potential of microglia by preventing release of TNF-α (50), whereas SLC1A5; a ferroptosis related gene has been correlated with recruitment and polarization of macrophages to anti-inflammatory M2 state (51). On the other hand, Tim-3 has been extensively investigated and shown to be expressed in glioma cells, MG, MACs, T, and NK cells with pro-tumorigenic characteristics (52). Its expression in TME is correlated with poor survival of glioma patients (53). Functionally, Tim-3 expression attenuates tumoricidal abilities of infiltrating T, NK cells, regulates polarization to anti-inflammatory M2 state, inhibits microglial phagocytosis (21) and promotes malignant behaviors of glioma cells (53). Although, our observations corroborate previous findings that MACs abundantly express Tim-3 in gliomas (54), but our results do not suggest increased Tim-3^+^MACs in IDH-wt compared to IDH-mutant gliomas unlike previously reported (54). Instead, we observed proportionately comparable levels of Tim-3^+^MACs regardless of IDH mutation status and tumor recurrence.

Because of abundant Galectin-9^+^ MG in human gliomas compared to non-glioma controls, we focused our attention to its intrinsic function. Like any other galectins, cytosolic, secreted and cell surface Galectin-9 protein may lead to pleiotropic functional outcomes, which could be difficult to discern *in vivo* (45). The prognostic value of Galectin-9 expression in glioma tissue remains debatable with gene expression correlated with shorter survival and protein expression not correlated with survival of GBM patients (39, 40). In this study, using human glioma tissues, we found that whereas flow cytometry revealed surface expression of Galectin-9 in MG, immunohistochemistry confirmed its total protein expression. Furthermore, our results demonstrate that Galectin-9 protein is predominantly expressed in immune cells, in particular GAMs, rather than in tumor cells contrary to previous reports in human gliomas (39). This discrepancy could be likely be attributed to differences in marker selection, for instance, the use of Olig2 in a previous study (39), and Nestin in this current study. Genetic variations in glioma cells, such as correlation of PTEN deficiency and Galectin-9 secretion likely offers explanation to discrepancies of Galectin-9 expression in tumor cells (33).

The cell autonomous function of Galectin-9 in DCs, MACs, Kupffer cells, and neutrophils has been reported as an important “eat me signal” that mediate phagocytosis of zymosan particles and cancer cells (55–59). In GBM, exosomal Galectin-9 bound to DC Tim-3 receptor inhibits antigen presentation and consequent T cell–mediated anti-tumor response (60). Soluble Galectin-9 acts as opsonin and together with VISTA can potentiate T-cell immunosuppressive effects in GBM (61). Overall, several studies point to targeting Galectin-9 in a variety of malignancies for relieving T-cell immune suppressive effects and augmenting phagocytosis (62). However, in lymphopenic cancers such as GBM, where TME is T and NK cell scarce, the trans effect of Galectin-9 on these cytotoxic cells may be inconsequential. Unlike, murine GL261 experimental GBM model which expressed Galectin-9 (34), we did not observe its expression in patient-derived tumor cells and GSCs that pathologically and immunogenically are more relevant disease models. In a GL261 study, murine Galectin-9^lo/-^ MG has been demonstrated to be pro-phagocytic than Galectin-9^hi/+^ MG, whereas our results suggest that human glioma-associated Galectin-9^+^ MG are enriched with phagocytic pathway genes compared to Galectin-9^-^ MG. Furthermore, inhibiting Galectin-9 have been reported to modestly improve survival and restrict tumor growth in mouse experimental GBM models (33, 34), a clinical utility that needs to be addressed in human settings. Our observation that Galectin-9 enhances phagocytosis by human microglial cells differs from recent murine glioma studies in which Galectin-9^hi^ microglia exhibited reduced phagocytic capacity and Galectin-9 blockade limited tumor growth (34). These differences are likely attributable, at least in part, to well-recognized species-specific differences in microglial biology. Comparative transcriptomic analyses have shown that human and mouse microglia diverge substantially in immune sensing and phagocytic pathways, which can lead to distinct functional outcomes in response to the same regulatory signals (63). In addition, Galectin-9 itself is not functionally identical across species; human and murine Galectin-9 differ in glycan-binding characteristics and receptor interactions, including TIM-3 engagement, resulting in divergent downstream signaling (64). Together, these species-specific differences, combined with the use of *in-vivo* murine tumor models versus human microglia–glioma stem-like cell co-cultures, likely contribute to the contrasting effects of Galectin-9 on microglial phagocytosis observed in our and other studies.

In this study, we adopted a reductionist approach and utilized simplified human-derived *ex-vivo* co-culture systems of pMG and GSCs to dissect intrinsic microglial functions independent of T cell– and NK cell–mediated effects. Downregulation of Galectin-9 gene or blocking the protein, both attenuated pMG adhesion and consequent phagocytosis. Our results highlight the cis acting role of Galectin-9 on pMG. As Galectin-9 protein downregulation was achieved by neutralizing antibody, which neutralizes the receptor, our observations indicate that modulating surface Galectin-9 is sufficient to potentiate phagocytosis in MG. Our study provides the much-needed evidence in human model system for glioma disease that Galectin-9 in MG facilitates phagocytosis of live tumor cells (termed as phagoptosis). Thus, lending support for Galectin-9 as a pro-phagocytic modulator, which can be therapeutically modulated for developing next-generation phagocytosis targeted GBM immunotherapies.

Although beyond the scope of current investigations, we acknowledge limitations associated with this study as enlisted herein; (i) we chose CellPhoneDB as the preferred tool for inferring L-R interactions despite its inherent caveats that relies on discrete L-R pairs and exclusion of non-peptide ligands, which has been included in the latest toolkit (65). This may have led to underestimation of key Galectin-9 interacting partners and molecular hubs beyond Galectin-9 operational in gliomas. (ii) Molecular mechanisms by which Galectin-9 mediates cell adhesion and phagocytosis needs to be dissected. (iii) Although we observed Galectin-9 expression in glioma-associated MDMs, we did not evaluate whether phagocytic functions of Galectin-9 are conserved even with infiltrating phagocytes akin to MG. (iv) *In-vivo* relevance of microglia-specific deletion of Galectin-9 in patient-derived xenograft mouse models. These pertinent questions need to be carefully assessed in future studies to harness, and reverse translate the phagocytic biology of Galectin-9.

Considering the aforesaid unresolved research questions, we suggest the following future research directions. (i) Given that inferring L-R interactions with single-cell data remains a challenging endeavor, corroborating our results with the latest version of CellPhoneDB (65) and complementary tools such as CellChatDB (66), ICELLNET (67), and likewise may help curate an exhaustive leukocyte interactome map that includes neuroendocrine and non-peptide L-R pairs. Using multiple bioinformatics toolkit simultaneously and a subsequent comparative approach that focuses on Galectin-9 associated L-R pairs will help identify its role within global cell communication networks and potential synergistic pathways. (ii) Since we identified Galectin-9–associated adhesion and phagosome factors, we suggest prioritizing phospho-proteomic analyses of putative molecular mediators (e.g., integrin-Rac axis) and direct protein interaction studies between Galectin-9 and phagosome assembly factors (e.g., coronin 1A) to dissect Galectin-9–mediated regulatory mechanisms that contribute to immunobiology of tumor cell adhesion and phagocytosis. (iii) Although it is challenging to discern between monocytic derivatives and macrophages in the glioma microenvironment, for which we have provided evidence of CCR2 positivity as a proxy to distinguish between monocytic origins and resident macrophages (2), we suggest evaluating Galectin-9 functions in blood monocytes to confirm functional conservation across cells of the phagocyte family. (iv) Due to different degree of aggressiveness of GSCs, *in-vivo* functional relevance of Galectin-9 should be studied in GSCs bearing xenograft mouse models mimicking multiple facets of glioma pathology such as from proneural to mesenchymal tumors by both loss and gain of function approach.

## 5 Conclusions

In summary, this human study provides novel insights into the immunobiology of Galectin-9 in GBM. By identifying a role for Galectin-9 in phagocytic functions, our study highlights important considerations for Galectin-9 targeted therapies, including the potential for unintended adverse effects on tumor-associated phagocytosis within the microenvironment. Based on our results, we suggest deploying Galectin-9 agonist or engineering Galectin-9 as a pro-phagocytic receptor, paving the way for novel GBM immunotherapeutic strategy.

## Data Availability

The datasets presented in this study can be found in online repositories. The names of the repository/repositories and accession number(s) can be found below: https://www.ncbi.nlm.nih.gov/, GSE222522.
