# Supplementary material for: Interrogation of glioma immune microenvironment identifies a non-canonical role for microglial Galectin-9 in tumor cell adhesion and phagocytosis
Source: Front Immunol. 2026 Mar 24;17:1733688. doi: 10.3389/fimmu.2026.1733688 (PMC13053254; doi:10.3389/fimmu.2026.1733688)
Supplement: Supplementary file 1 [file DataSheet1.docx]

Supplementary Material

**Supplementary Fig. S1**

**Supplementary Fig. S1.** (related to Fig. 2). Expression of ligand (Galectin-9/PD-L1) and cognate receptors (Tim-3/PD-1) in glioma-associated leukocytes. A-B, Representative FCM contour plots show the expression of Galectin-9, PD-L1, Tim-3 and PD-1 on myeloid lineage cell types; MG, MAC, MDM, cDC1 and, cDC2 in (A) and Tim-3 and PD-1 in lymphoid lineage cells; CD4^+^ T, CD8^+^ T and NK cells in (B) of glioma associated leukocytes isolated from patients. C-D, Corresponding, color-coded scatter bar plots represent the relative proportions of PD-L1^+^ and PD-1^+^ cells of indicated myeloid populations in (C) and lymphoid populations in (D) across glioma subtypes as shown. E-F, Corresponding, color-coded scatter bar plots represent the relative proportions of PD-L1^+^Galectin-9^+^ and PD-1^+^Tim-3^+^ cells of indicated myeloid cells in (E) and PD-1^+^Tim-3^+^ lymphoid populations in (F) across glioma subtypes as shown. NGB (n = 3), IMP (n = 14), IMR (n = 9), IWP (n = 13), IWR (n = 12). Statistical differences were determined using Kruskal-Wallis test followed by Dunn’s post hoc test for multiple comparisons at indicated p values between NGB vs glioma subtypes, IMP vs IMR, IWP vs IWR, and IMP vs IWP. n.s.= statistically not significant.

**Supplementary Fig. S2**

**
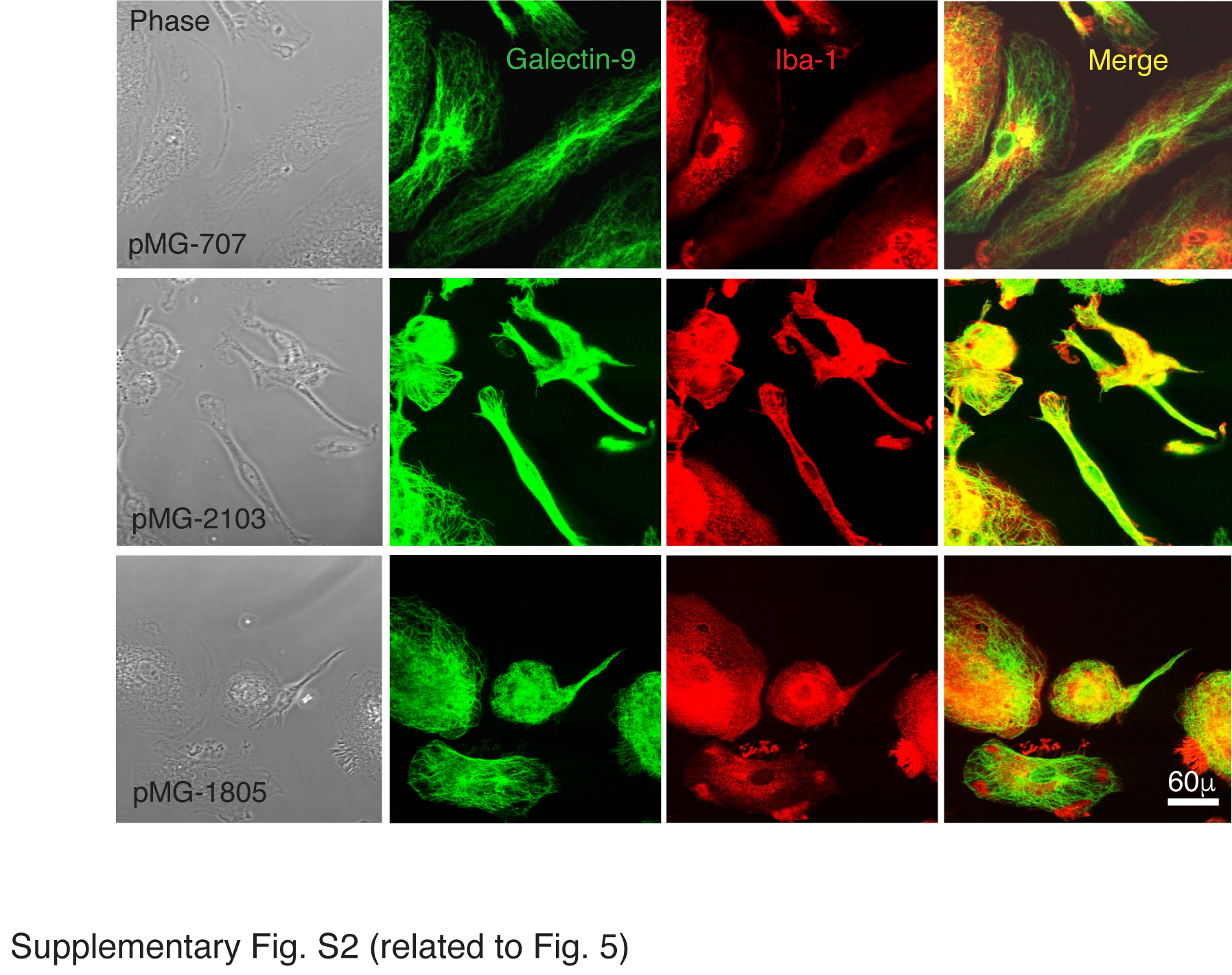
**

**Supplementary Fig. S2** (related to Fig. 5). Galectin-9 and Iba-1 expression on pMG. Representative microscopic immunofluorescence image showing phase contrast view and staining of Galectin-9 (green) and Iba-1 (red) and their composite expression in merged image in pMGs from three different fetal donors (pMG-707, pMG-2103, pMG-1805). Scale bars = 60 μm.

**Supplementary Fig. S3**


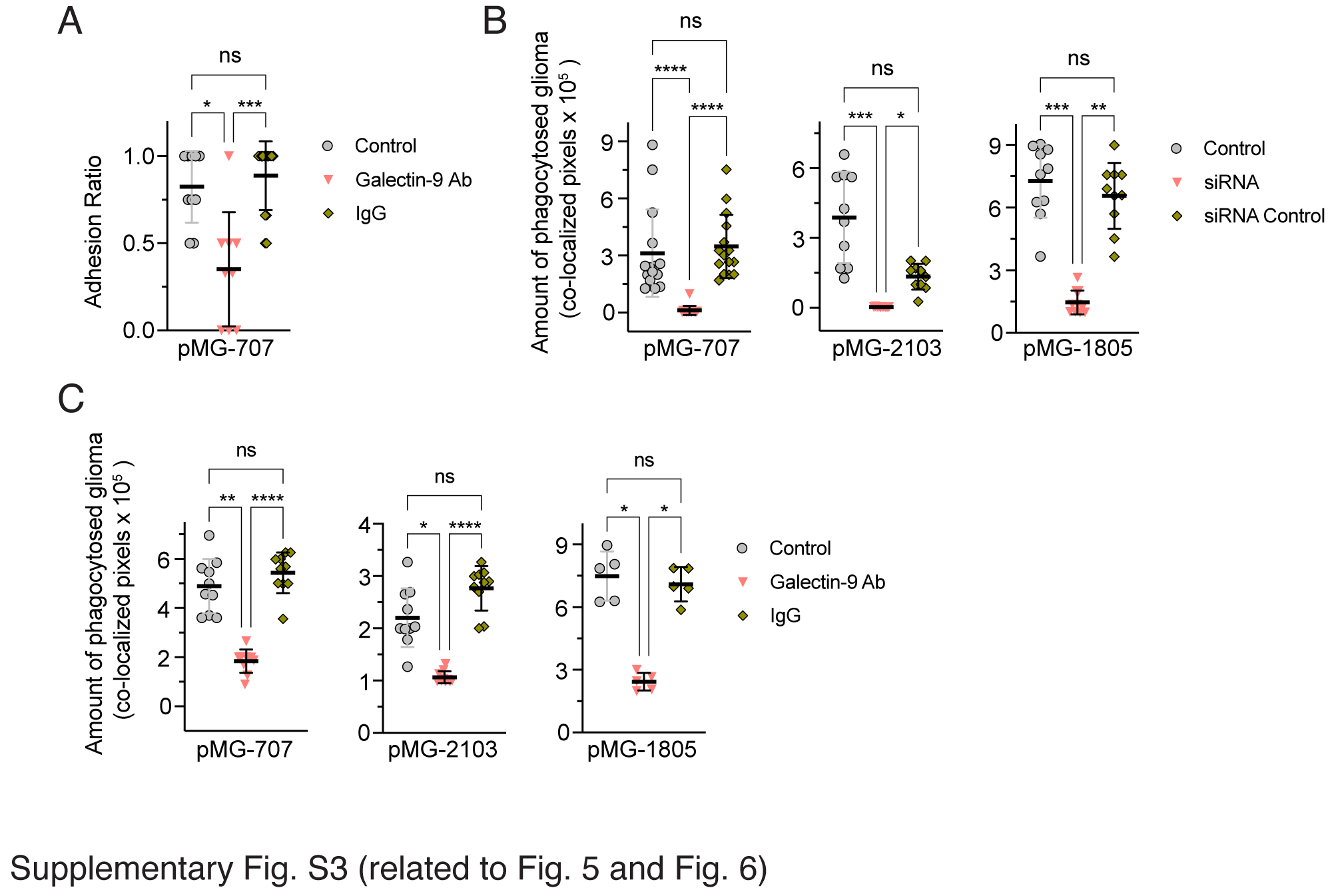


**Supplementary Fig. S3** (related to Fig. 5 and 6). Adhesion ratio and phagocytic uptake of pMGs. A, Scatter dot plots showing proportions as mean +/− SD of % GSC8-11ZsG adhered to pMG-707 (adhesion ratio) in untreated control, treated Galectin-9 siRNA and siRNA control experimental conditions. Error bars indicate the SD of mean. Statistical differences were determined using Kruskal-Wallis test followed by Dunn’s post hoc test for multiple comparisons between siRNA treated vs control groups at *p<0.05, ***p<0.001. B, Scatter dot plots showing amount of phagocytosed GSC8-11ZsG represented as mean value colocalized pixels +/− SD of pMGs (pMG-2103, pMG-707 and pMG-1805) that phagocytosed GSC8-11ZsG in untreated control, treated Galectin-9 siRNA and siRNA control experimental conditions when co-cultured with GSC8-11ZsG. Error bars indicate the SD of mean. statistical differences were determined using Kruskal-Wallis test followed by Dunn’s post hoc test for multiple comparisons between siRNA treated vs control groups at *p<0.05, **p<0.01, ****p<0.001, ****p<0.0001. C, Scatter dot plots showing amount of phagocytosed GSC8-11ZsG represented as mean value of colocalized pixels +/− SD of pMGs (pMG-2103, pMG-707 and pMG-1805) that phagocytosed GSC8-11ZsG in untreated control, treated Galectin-9 neutralization Ab (MAb-13) and IgG control experimental conditions when co-cultured with GSC8-11ZsG. Error bars indicate the SD of mean. Statistical differences were determined using Kruskal-Wallis test followed by Dunn’s post hoc test for multiple comparisons between siRNA treated vs control groups at *p<0.05, **p<0.01, ****p<0.0001.
